# Mapping the genetic architecture of low‐temperature stress tolerance in citron watermelon

**DOI:** 10.1002/tpg2.20443

**Published:** 2024-03-10

**Authors:** Dennis N. Katuuramu, Amnon Levi, William P. Wechter

**Affiliations:** ^1^ USDS‐ARS, U.S. Vegetable Laboratory Charleston South Carolina USA

## Abstract

Sweet‐fleshed watermelon (*Citrullus lanatus*) is an important vegetable crop of the tropical origin. It is widely grown and consumed around the world for its hydration and nutritional quality values. Low‐temperature stress can affect early planting, seedling establishment, and expansion of crop production to new areas. A collection of 122 citron watermelon (*Citrullus amarus*) accessions were obtained from the USDA's National Plant Germplasm Repository System gene bank in Griffin, GA. The accessions were genotyped using whole genome resequencing to generate single nucleotide polymorphisms (SNPs) molecular markers and screened under cold‐stressed and non‐stressed control conditions. Four low‐temperature stress tolerance related traits including shoot biomass, vine length, maximum quantum efficiency of photosystem II, and chlorophyll content were measured under cold‐stressed and non‐stressed control treatment conditions. Correlation analysis revealed the presence of positive relationships among traits. Broad‐sense heritability for all traits ranged from 0.35 to 0.73, implying the presence of genetic contributions to the observed phenotypic variation. Genomic regions underlying these traits across several citron watermelon chromosomes were identified. Four low‐temperature stress tolerance related putative candidate genes co‐located with the peak SNPs from genome‐wide association study. These genomic regions and marker information could potentially be used in molecular breeding to accelerate genetic improvements for low‐temperature stress tolerance in watermelon.

## Core Ideas

- A panel of citron watermelon accessions was extensively phenotyped for cold stress tolerance related traits under both stressed and non‐stressed control conditions.
- Using 122 accessions and 2,126,759 genome‐wide single nucleotide polymorphism markers, genome‐wide association study models were evaluated to discover marker‐trait associations.
- Genomic loci and candidate genes were identified and will be useful in the improvement of watermelon varieties for cold stress tolerance traits.

**Abbreviations**
BLINK: Bayesian‐information and linkage‐disequilibrium iteratively nested keyway
BLUE: best linear unbiased estimate
FarmCPU: fixed and random model circulating probability unification
GWAS: genome‐wide association study
QQ: quantile–quantile
REML: restricted maximum likelihood
SNP: single nucleotide polymorphism
SPAD: soil and plant analyzer development

## 1 INTRODUCTION

Sweet‐fleshed watermelon (*Citrullus lanatus*) is an economically important vegetable crop that originated from tropical Africa and is currently grown in both temperate and tropical regions around the world (Chomicki et al., 2020; Chomicki & Renner, 2015). Globally, the crop is planted on a land area of more than 3 million ha with production exceeding 100 million tonnes per year (FAOSTAT, 2022). Citron watermelon (*Citrullus amarus*) is also an important member of the *Cucurbitaceae* family that originated from tropical southern Africa. *Citrullus amarus* crosses readily with *C*. *lanatus* and can thus be utilized to improve the cultivated watermelon through controlled pollinations (Chomicki et al., 2020; Chomicki & Renner, 2015; Jarret et al., 2017; Levi et al., 2013, 2001). Watermelon is sensitive to low temperatures especially during early growth stages in the production season of temperate regions (Bradow, 1990). The optimum environmental temperature for effective watermelon plant growth varies from 18 to 35°C, where any temperature outside this range will slow down growth, maturation, and reduce crop yield (Korkmaz & Dufault, 2001). Transplanting of watermelon seedlings in temperate regions is often delayed to mid to late spring (April to mid‐May) when temperatures are warmer with minimal risks of plant death from low temperatures (Boyhan et al., 2000; Egel et al., 2008; NeSmith, 1999). In the United States, maximum consumption and premium prices for watermelon fruits occurs from June to mid‐July (Kaiser, 2012; https://www.ers.usda.gov/). For the watermelon growers to maximize profits during these target dates, low‐temperature tolerant varieties that can be transplanted earlier in the growing season need to be developed.

Low temperature early in the growing season is a major impediment to plant establishment, growth, and development, especially for plant species of tropical origin including watermelon (Cramer et al., 2001). Generally, when plants of tropical origin are exposed to low temperatures (<10°C), they incur cellular and tissue injuries, reduced chlorophyll synthesis and content, reduction in photosynthetic rate including photosystem II efficiency, decreased biomass synthesis, diminished vigor, disruption in water relations, and hormonal imbalance (Knight & Knight, 2012; Revilla et al., 2005; Shinozaki et al., 2015). Moreover, exposure of young watermelon seedlings to low temperatures can result in stunted growth, necrotic lesions and burns on the leaves, increased susceptibility to pests and diseases, and yield reductions (Bradow, 1990; Korkmaz & Dufault, 2001). Improved low‐temperature stress tolerance in watermelon will allow for earlier transplanting dates, escape of summer heat and drought, a reduction in pests and disease pressures, as well as fruit maturity dates that coincide with months of highest fruit demand and premium prices.

Recent advances in watermelon genomics have led to the development of several genomic resources including annotated genomes and single nucleotide polymorphism (SNP) molecular marker datasets for both cultivated and citron watermelons (Guo et al., 2019; Katuuramu et al., 2022; Wu et al., 2019; http://cucurbitgenomics.org/). These genome analysis efforts have enabled the dissection of the genetic control of various traits in watermelon using several mapping strategies such as genome‐wide association study (GWAS). GWAS utilizes diversity panels that have been densely genotyped with SNP markers and phenotyped for traits of interest to detect marker‐trait associations. The GWAS methodology relies on linkage disequilibrium patterns and historical recombination present in diverse germplasm to uncover genomic regions controlling important traits in plants (Ingvarsson & Street, 2011; Zhu et al., 2008). GWAS has recently been deployed to detect genomic regions underlying downy mildew resistance, flowering sex expression, fruit yield components, seed chemical composition, and fruit quality traits in watermelon (Aguado et al., 2020; Joshi et al., 2021; Katuuramu et al., 2023a, 2022, 2023b).

There is limited information and research data on the genetic control for low‐temperature stress tolerance in watermelon. A couple of studies have focused on screening method optimization and worked with small sets of watermelon genotypes in such trials (Kozik & Wehner, 2014; Korkmaz & Dufault, 2002). Research reports in cucumber and cantaloupes have shown that low‐temperature stress tolerance is a complex trait controlled by multiple genes. Previous studies have revealed multiple quantitative trait loci controlling tolerance to low‐temperature stress in cucumber and cantaloupes using recombinant inbred line mapping populations (Hou et al., 2018; Song et al., 2018; Yagcioglu et al., 2019). Currently, there are no published research reports on the efforts to decipher the genetic architecture of low‐temperature stress tolerance in watermelon. The main objectives of this study were to evaluate low‐temperature stress tolerance related traits across the 122 citron watermelon genotypes exposed to a temperature of 4°C. Trait performance was evaluated on the entire GWAS panel subjected to both cold‐stressed and non‐stressed control conditions in the growth chamber. Second, using the traits and genotypic datasets, GWAS was performed to gain insights into the genetic control of low‐temperature stress tolerance in citron watermelon.

## 2 MATERIALS AND METHODS

### 2.1 Plant material, growth chamber conditions, and experimental design

A total of 122 *C*. *amarus* plant introduction accessions were evaluated for low‐temperature tolerance. The accessions were originally received from the USDA National Plant Germplasm System in Griffin, GA, and have been described in previous research efforts (Katuuramu et al., 2022). Seeds for each accession were sown in 36‐cell vegetable propagation trays (T.O. Plastics Inc.) filled with Metro‐Mix 360 (Sun Gro Horticulture). To ensure optimum growth, the seedlings were watered as needed, while fertilizer, growth temperature, light source, and photo‐period regime were as described previously by Katuuramu et al. (2022). The seedlings were grown for 4 weeks post‐seeding before initiation of low‐temperature stress of 4°C that lasted for 24 h in a climate‐controlled growth chamber (Kozik & Wehner, 2014). After the 24‐h low‐temperature stress, the seedlings were allowed to recover for 7 days prior to assessment of the low‐temperature stress related traits. The low‐temperature stress intensity of 4°C and duration of 24 h were chosen based on a previous watermelon study (Kozik & Wehner, 2014). Additionally, plants were stressed at the 4 weeks post‐seeding developmental stage because in watermelon production, growers transplant seedlings to the outside open‐fields 4 weeks following seeding (Olson et al., 1994; Vavrina et al., 1993). This low‐temperature tolerance research work for both cold‐stressed and non‐stressed control experiments was conducted as a randomized complete block design with two replications over two screening tests.

**Core Ideas**

- A panel of citron watermelon accessions was extensively phenotyped for cold stress tolerance related traits under both stressed and non‐stressed control conditions.
- Using 122 accessions and 2,126,759 genome‐wide single nucleotide polymorphism markers, genome‐wide association study models were evaluated to discover marker‐trait associations.
- Genomic loci and candidate genes were identified and will be useful in the improvement of watermelon varieties for cold stress tolerance traits.

### 2.2 Traits measurement

For both cold‐stressed and non‐stressed control plants, trait data collection was at 5 weeks post‐seeding. One week after completion of the cold treatment, four low‐temperature stress related traits were evaluated: 1) maximum quantum efficiency of photosystem II (*F_v_*/*F_m_*, or variable fluorescence divided by maximum fluorescence), where variable fluorescence is the difference between maximum fluorescence and minimum fluorescence (*F_o_*). The *F_v_*/*F_m_* values were measured on the uppermost fully expanded leaf using a handheld chlorophyll fluorometer (OS30p+, Opti‐Sciences). Prior to each measurement, a 9.53‐mm diameter dark clip with a light excluding shutter was attached to the upper leaf surface of every plant for at least 2 h to render the surface dark‐adapted and induce maximal possible *F_m_* values in closed photosystem II reaction centers (Murchie & Lawson, 2013). To take *F_v_*/*F_m_* measurements, the chlorophyll fluorometer was set to a saturation pulse width of 1 s and a saturation intensity of 6000 µmol m^−2^ s^−1^. 2) Relative leaf chlorophyll content in soil and plant analyzer development (SPAD) units of the uppermost fully expanded leaf was measured using a handheld chlorophyll meter (SPAD, SPAD‐502, Minolta). For every plant, SPAD values were derived from the average of two readings from the base and tip of the uppermost fully expanded leaf. 3) Fresh shoot biomass was assessed by cutting vines at the soil‐vine junction and weighing the cut vines in grams per plant. 4) To assess vine length, cut vines for every genotype were photographed with a Sony Cyber‐shot DSC‐HX400V camera equipped with a 24–1200 mm ZEISS lens (SONY). Photographs (red–green–blue images) were then processed using Fiji (ImageJ) software to extract vine length in mm (Schindelin et al., 2012).

### 2.3 Statistical data analyses

Phenotypic data analysis was performed using the statistical analysis software (SAS v9.4) and R v4.2.0 programming language (R Core Team, 2022; SAS Institute, 2013). Analysis of variance (ANOVA) was performed in SAS using the MIXED procedure (SAS Institute, 2013). For all low‐temperature tolerance traits, the statistical model included genotype, screening test, interaction term for genotype and screening test, and replication nested in screening test. To generate ANOVA, effects of genotype were classified as fixed, while the rest of the model components were random. To compare genotype performance for every low‐temperature stress related trait, mean separation was performed using Fisher's protected least significant difference at an *α* level of 0.05 in SAS v9.4 (SAS Institute, 2013). Welch's two‐sided *t*‐test was implemented in R software for statistical comparisons between the cold‐stressed and non‐stressed control citron watermelon genotype pools (R Core Team, 2022). Variance components for every trait were generated using the VARCOMP procedure in SAS v9.4 using the restricted maximum likelihood (REML) estimation method (SAS Institute, 2013). Broad‐sense heritability on an entry‐mean basis for every trait was computed as described by Holland et al. (2003) and the following formula: Var(*G*)/[Var(*G*) + Var(*G* × *T*)/*t* + Var(Error)/*tr*], where Var is variance, G is genotype, *G* × *T* is genotype by growth chamber screening test interaction, and *t* and *r* are the number of growth chamber screening tests and replications, respectively. Pearson correlations among traits were computed using PROC CORR in SAS v9.4 (SAS Institute, 2013).

### 2.4 Genotyping and GWAS analysis

Methods for genomic DNA isolation, sequencing technologies, and SNP variant calling were described previously by Katuuramu et al. (2022). Briefly, whole‐genome resequencing data for the 122 citron watermelon (*C*. *amarus*) accessions were obtained using Illumina NovaSeq 6000 sequencing technology (Illumina). Shotgun genomic libraries were sequenced on a single lane using the Illumina NovaSeq 6000 machine to generate 150 bp paired‐end reads. Reads were filtered and trimmed as presented previously in Katuuramu et al. (2022). Variant discovery and quality control were conducted using the GATK v3.6 best practices workflow (Depristo et al., 2011; McKenna et al., 2010; van der Auwera et al., 2013). After removal of markers with minor allele frequency <0.05, a total of 2,126,759 SNPs were retained for downstream analyses. GWAS for the evaluated traits was performed using GAPIT v3.0 implemented in R v4.2.0 (Lipka et al., 2012; R Core Team, 2022; J. Wang & Zhang, 2021). Phenotypic data were converted to best linear unbiased estimates (BLUEs) using the REML method to minimize shrinkage toward the population mean and implemented in SAS v9.4 (SAS Institute, 2013). The BLUEs were then used as the trait input files in GAPIT v3.0 to perform GWAS. Population structure and kinship were assessed in GAPIT v3.0 within R v4.2.0 (Lipka et al., 2012; R Core Team, 2022; Wang & Zhang, 2021). The VanRaden (2008) method was used to create a kinship matrix in GAPIT v3.0. Principal components (PCs) to account for population stratification during GWAS were generated using the *prcomp* function in R v4.2.0 (R Core Team, 2022). The optimum number of PCs to include as covariates for every trait was determined using scree plot analysis and Bayesian information criterion‐based selection procedure in GAPIT v3.0 (Schwarz, 1978).

To select the optimal GWAS model, four models were evaluated: general linear model or GLM (only accounts for population structure); mixed linear model or MLM (accounts for population structure and cryptic relatedness); fixed and random model circulating probability unification (FarmCPU); and Bayesian‐information and linkage disequilibrium iteratively nested keyway, or BLINK (Huang et al., 2018; Liu et al., 2016; Yu et al., 2006). Both BLINK and FarmCPU are multi‐locus models and were chosen as the preferred methods to detect associations between the SNPs and traits. BLINK and FarmCPU were the models of choice for this research because they have increased statistical power, are computationally efficient for big genotypic datasets with millions of SNP markers, and better control both false positives and false negatives compared to the preceding single‐locus models such as GLM and MLM (Huang et al., 2018; Liu et al., 2016; Yu et al., 2006). Significance levels for multiple marker‐trait association testing were established using the false discovery rate at a 0.05 *α* value (Benjamin & Hochberg, 1995). The likelihood ratio based *R* ^2^ statistic was used to calculate the phenotypic variation explained by the peak SNP markers for every trait (Sun et al., 2010). Allelic effects of the peak SNPs on trait performance were compared using a two‐sided distribution Welch's *t*‐test and visualized in R v4.2.0 (R Core Team, 2022). Manhattan and quantile–quantile (QQ) plots to visualize marker‐trait associations and goodness of model fitting were generated using the *CMplot* package in R v4.2.0 software (Yin, 2022). Model‐trait associations exhibiting irregular deviations in QQ plots are not presented.

### 2.5 Candidate gene search

Putative candidate genes across the annotated USVL246‐FR2 reference genome were detected using search windows presented previously in Katuuramu et al. (2022). Plausible candidate genes included those whose description(s) and gene ontology functions were relevant to molecular low‐temperature stress tolerance, biomass accumulation, and photosynthesis. Also, a literature search was performed to find orthologs and functional gene data in other crop systems with similarity to candidate genes uncovered in this research.

## 3 RESULTS

### 3.1 Descriptive summary statistics, broad‐sense heritability, and correlation analysis

**FIGURE 1:**
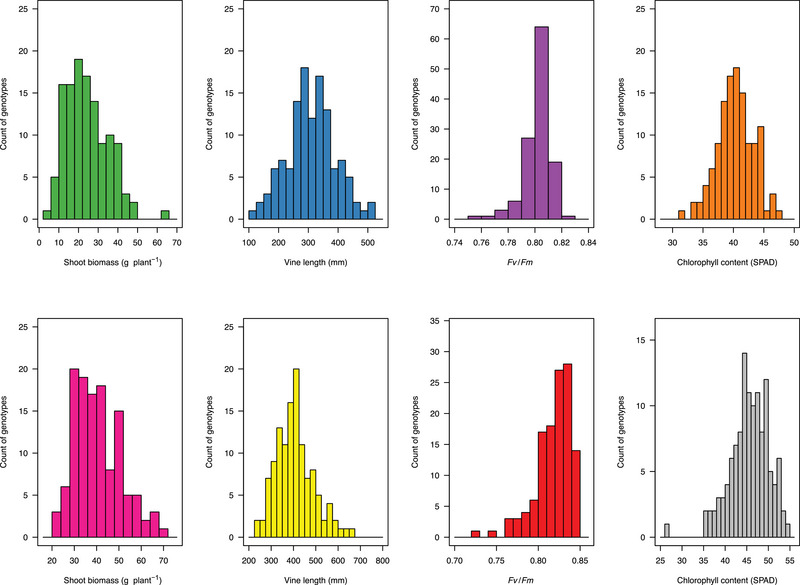
Histogram showing distribution of the evaluated traits including shoot biomass, vine length, *F_v_*/*F_m_*, and chlorophyll content across the 122 citron watermelon seedlings averaged over two screening tests. Top row represents trait distribution for the cold‐stressed plants. Bottom row shows trait histograms for the non‐stressed control plants. SPAD, soil and plant analyzer development.

There were significant differences (*p* ≤ 0.001) among the citron watermelon genotypes evaluated for low‐temperature stress tolerance in this research. Phenotypic variation for shoot biomass averaged over two screening tests ranged from 3.1 to 65.1 g plant^−1^ with a fold variation of 21 (Table 1; Figure 1). Vine length ranged from 114.2 to 516.1 mm and had 4.5‐fold variation (Table 1; Figure 1). *F_v_*/*F_m_* ranged from 0.76 to 0.82 with a fold variation of 1.1 (Table 1; Figure 1). Chlorophyll content ranged from 31.9 to 48 SPAD units with a fold variation of 1.5 (Table 1; Figure 1).

**TABLE 1:** Phenotypic summary statistics with mean, standard deviation (SD), range (minimum and maximum), *t*‐test, and broad‐sense heritability (*H* ^2^) values of the four traits for all the 122 citron watermelon seedlings evaluated under cold stress and non‐stress control conditions over two screening tests conducted in the growth chamber.

Traits	Cold‐stressed		Non‐stressed control		*df*	*t* value	*p* value	*H* ^2^
	Mean ± SD	Range	Mean ± SD	Range				
Shoot biomass (g plant^−1^)	24.3 ± 10.7	3.1–65.1	40.4 ± 10.3	21.8–71.8	241.7	−11.95	3.6 × 10^−26^	0.57
Vine length (mm)	309.6 ± 80.4	114.2–516.1	405.4 ± 84.0	235.8–659.6	241.5	−9.10	3.4 × 10^−17^	0.73
*F_v_*/*F_m_*	0.80 ± 0.01	0.76–0.82	0.82 ± 0.02	0.72–0.85	170.3	−0.48	ns	0.42
Chlorophyll content (SPAD)	40.4 ± 3.0	31.9–48.0	45.6 ± 4.5	26.8–54.6	209.2	−10.70	1.3 × 10^−21^	0.35

Abbreviations: *df*, degrees of freedom; ns, not significant; SPAD, soil and plant analyzer development.

**FIGURE 2:**
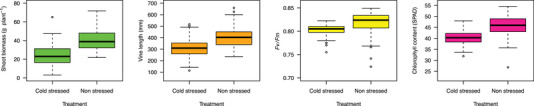
Boxplot showing the effect of cold stress versus non‐stress control treatments on the evaluated traits including shoot biomass, vine length, *F_v_*/*F_m_*, and chlorophyll content across the 122 citron watermelon seedlings averaged over two screening tests. SPAD, soil and plant analyzer development.

Under non‐stress control treatment conditions, shoot biomass ranged from 21.8 to 71.8 g plant^−1^ with a fold variation of 3.3 (Table 1; Figure 1). Vine length varied from 235.8 to 659.6 mm and had 2.8‐fold variation (Table 1; Figure 1). *F_v_*/*F_m_* varied from 0.72 to 0.85 with a fold variation of 1.2 (Table 1; Figure 1). Chlorophyll content ranged from 26.8 to 54.6 SPAD units with a fold variation of 2.0 (Table 1; Figure 1). Overall, Welch's two‐sided *t*‐test analysis revealed lower trait means within the cold‐stressed plants compared to the non‐stressed control plants (Table 1; Figure 2). For shoot biomass, cold‐stressed plants weighed 24.3 g plant^−1^ compared to 40.4 g plant^−1^ among the non‐stressed control plants (Table 1; Figure 2). Cold‐stressed plants had shorter vine length (mean of 309.6 mm) compared to 405.4 mm among the non‐stressed control plants (Table 1; Figure 2). There was no statistical difference between the mean *F_v_*/*F_m_* of cold‐stressed versus non‐stressed control plants (Table 1; Figure 2). The non‐stressed control plants had higher chlorophyll content (mean of 45.6 SPAD units) compared to cold‐stressed plants with a mean of 40.4 SPAD units (Table 1; Figure 2). Broad sense heritability for the low‐temperature stress tolerance related traits ranged from 0.35 to 0.73 (Table 1). Shoot biomass and vine length had moderately high broad sense heritability of 0.57 and 0.73, respectively (Table 1). Chlorophyll content and *F_v_*/*F_m_* had moderately low heritability of 0.35 and 0.42, respectively (Table 1).

Genotypes USVL_114 and PI_532664 were part of the top five accessions for shoot biomass with 65.1 and 44.2 g plant^−1^, respectively, under cold‐stressed conditions (Table 2). Genotypes PI_532664 and USVL_114 had the highest measurement for vine length of 516.1 and 501 mm, respectively (Table 2). Genotypes PI_271769 and PI_482265 had low values for both shoot biomass and vine length (Table 2). Genotype PI_271769 had the lowest shoot biomass of 3.1 g plant^−1^ and low *F_v_*/*F_m_* ratio of 0.77 (Table 2). Genotype PI_482257 had a low biomass of 6.1 g plant^−1^ and chlorophyll content of 33.7 SPAD units (Table 2).

**TABLE 2:** List of some of the superior genotypes from the 122 citron watermelon collection evaluated under low‐temperature stress over two screening tests conducted in the growth chamber.

Genotype ID	Country of origin	Continent	Shoot biomass (g plant^−1^)	Vine length (mm)	*F_v_*/*F_m_*	Chlorophyll content (SPAD)
USVL_114	Botswana	Africa	65.1	501.0	0.81	39.1
PI_482312	Zimbabwe	Africa	47.4	346.1	0.80	40.0
PI_532666	Eswatini	Africa	46.2	454.5	0.81	38.9
PI_606135	Russia	Europe	44.4	411.7	0.81	39.7
PI_532664	Eswatini	Africa	44.2	516.1	0.81	40.7
PI_542114	Botswana	Africa	39.7	491.1	0.80	37.1
PI_482246	Zimbabwe	Africa	39.7	350.9	0.81	46.4
PI_500335	Zambia	Africa	35.9	369.6	0.81	46.8
PI_596669	South Africa	Africa	28.3	473.3	0.79	44.9
PI_296339	South Africa	Africa	27.1	334.8	0.81	48.0
PI_296342	South Africa	Africa	24.9	392.6	0.81	34.4
PI_270564	South Africa	Africa	23.5	160.1	0.78	31.9
PI_596692	South Africa	Africa	22.6	358.4	0.82	43.5
PI_244019	South Africa	Africa	22.4	294.6	0.76	38.1
PI_271767	South Africa	Africa	20.7	429.3	0.82	38.1
PI_532668	Eswatini	Africa	18.4	276.4	0.82	40.3
PI_482319	Zimbabwe	Africa	18.2	334.2	0.82	46.1
PI_270563	South Africa	Africa	15.4	141.6	0.80	36.5
PI_596665	South Africa	Africa	14.2	249.3	0.82	40.3
PI_295843	South Africa	Africa	13.3	210.1	0.82	42.1
PI_296341	South Africa	Africa	10.6	191.3	0.81	34.0
PI_482342	Zimbabwe	Africa	10.1	203.0	0.78	40.2
PI_244018	South Africa	Africa	8.2	156.4	0.81	39.2
PI_596696	South Africa	Africa	7.7	239.8	0.80	36.6
PI_482265	Zimbabwe	Africa	6.1	142.5	0.81	38.1
PI_482265	Zimbabwe	Africa	6.1	142.5	0.81	38.1
PI_482257	Zimbabwe	Africa	6.1	165.6	0.80	33.7
PI_271769	South Africa	Africa	3.1	114.2	0.77	35.3
LSD (*α* = 0.05)			12.4	72.3	0.02	4.3

Abbreviations: LSD, least significant difference; SPAD, soil and plant analyzer development.

Correlation analysis revealed the presence of positive relationships among all trait combinations regardless of the stress treatment status (Table 3). For the cold‐stressed experiments, shoot biomass was highly corelated to vine length (*r* = 0.76) but with low correlation to *F_v_*/*F_m_* (*r* = 0.19). Vine length had a low correlation to *F_v_*/*F_m_* (*r* = 0.21) and chlorophyll content (*r* = 0.18). Chlorophyll content had a moderately low correlation to *F_v_*/*F_m_* of *r* = 0.29 (Table 3). For the non‐stressed control experiments, there was moderate correlation between vine length and shoot biomass (*r* = 0.50) (Table 3). There were no statistically significant correlations between *F_v_*/*F_m_* with shoot biomass or vine length (Table 3). Chlorophyll content had moderately low correlations with shoot biomass (*r* = 0.33) and *F_v_*/*F_m_* (*r* = 0.40) (Table 3).

**TABLE 3:** Phenotypic correlations among the evaluated traits under cold‐stressed and non‐stressed control conditions of the 122 citron watermelon seedlings grown in growth chamber.

Traits	Shoot biomass	Vine length	*F_v_*/*F_m_*	Chlorophyll content
Shoot biomass	–	0.76^**^	0.19^*^	0.16 ns
Vine length	0.50^**^	–	0.21^*^	0.18^*^
*F_v_*/*F_m_*	0.13 ns	0.01ns	–	0.29^**^
Chlorophyll content	0.33^**^	0.03ns	0.40^**^	–

*Note*: Upper diagonal represents correlations among traits for cold‐stressed citron watermelon plants; trait correlations for the non‐stressed control seedlings are provided in the lower diagonal.

^*^and **significant at *p* ≤ 0.05 and *p* ≤ 0.001 probability levels; ns, not significant.

### 3.2 Marker‐trait associations and allelic effects on the evaluated traits

**FIGURE 3:**
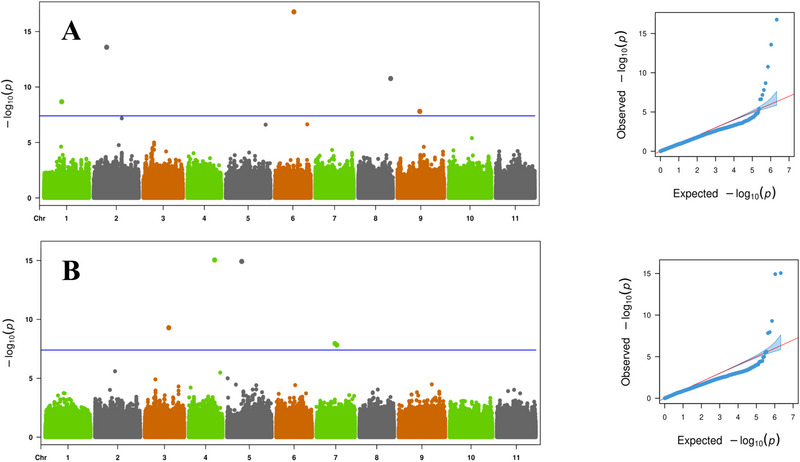
Manhattan and quantile–quantile (QQ) plots for the evaluated traits of (A) shoot biomass and (B) *F_v_/F_m_* based on the FarmCPU (fixed and random model circulating probability unification) model across the 122 cold‐stressed citron watermelon genotypes using best linear unbiased estimates (BLUEs) from the growth chamber screening. The blue solid horizontal line is a false discovery rate (FDR) cutoff of *α* = 0.05.

Significant SNP markers associated with the evaluated traits under cold stress and non‐stress control conditions were identified in this research (Table 4). Under cold stress conditions, five SNP markers were associated with shoot biomass (*p* value ≤ 1.6 × 10^−8^) and were located on chromosomes Ca01, Ca02, Ca06, Ca08, and Ca09 (Table 4; Figure 3A). All the five peak SNPs were uncovered by the FarmCPU model and explained 3.3%–16.6% of the phenotypic variation in shoot biomass (Table 4). There were five peak SNPs associated with *F_v_*/*F_m_* (*p* value ≤ 1.5 × 10^−8^) and were located on chromosomes Ca03, Ca04, Ca05, and Ca07 (Table 4; Figure 3B). All the five SNP markers were detected using the FarmCPU model and explained 2.1%–17.2% of the phenotypic variation in *F_v_*/*F_m_* (Table 4).

**TABLE 4:** Details of the peak single nucleotide polymorphisms (SNPs) associated with shoot biomass, *F_v_*/*F_m_*, and vine length traits across the cold‐stressed and non‐stressed control 122 citron watermelon seedlings based on BLINK (Bayesian‐information and linkage‐disequilibrium iteratively nested keyway) and FarmCPU GWAS, where FarmCPU is fixed and random model circulating probability unification and GWAS is genome‐wide association study, models.

Traits	Treatment	Chr	SNP	SNP position (bp)	SNP *p* value	MAF	Major allele	Minor allele	PVE (%)	GWAS model
Shoot biomass	Cold‐stressed	Ca01	S1_14771716	14,771,716	2.1E‐09	0.07	C	A	5.6	FarmCPU
		Ca02	S2_10773112	10,773,112	2.6E‐14	0.34	C	T	4.1	FarmCPU
		Ca06	S6_15816015	15,816,015	1.7E‐17	0.15	C	T	16.6	FarmCPU
		Ca08	S8_26904287	26,904,287	1.7E‐11	0.10	G	A	9.2	FarmCPU
		Ca09	S9_18593292	18,593,292	1.6E‐08	0.07	C	A	3.3	FarmCPU
*F_v_*/*F_m_*	Cold‐stressed	Ca03	S3_20011267	20,011,267	5.1E‐10	0.06	G	A	9.1	FarmCPU
		Ca04	S4_21563874	21,563,874	8.9E‐16	0.14	G	A	9.2	FarmCPU
		Ca05	S5_12205786	12,205,786	1.2E‐15	0.19	T	C	17.2	FarmCPU
		Ca07	S7_15365202	15,365,202	1.1E‐08	0.10	C	T	2.1	FarmCPU
		Ca07	S7_16889997	16,889,997	1.5E‐08	0.10	A	T	12.2	FarmCPU
	Non‐stressed	Ca02	S2_24000594	24,000,594	5.7E‐09	0.05	C	T	21.8	BLINK, FarmCPU
		Ca03	S3_31686848	31,686,848	2.3E‐08	0.09	T	A	13.7	BLINK, FarmCPU
Vine length	Cold‐stressed	Ca02	S2_12134590	12,134,590	2.5E‐13	0.27	T	C	12.3	BLINK, FarmCPU
		Ca05	S5_7369447	7,369,447	6.3E‐09	0.33	G	A	4.4	FarmCPU
		Ca06	S6_16004962	16,004,962	2.1E‐13	0.24	G	A	15.7	BLINK, FarmCPU
	Non‐stressed	Ca01	S1_451332	451,332	2.6E‐11	0.40	A	C	3.4	FarmCPU
		Ca05	S5_22850438	22,850,438	5.7E‐11	0.05	G	A	7.6	FarmCPU
		Ca05	S5_28595908	28,595,908	2.1E‐08	0.19	A	C	8.8	FarmCPU
		Ca05	S5_30282239	30,282,239	9.0E‐09	0.48	T	C	2.5	FarmCPU
		Ca06	S6_1262616	1,262,616	9.9E‐09	0.31	C	G	5.0	FarmCPU
		Ca06	S6_18850800	18,850,800	2.6E‐10	0.49	C	T	3.6	FarmCPU
		Ca09	S9_16767943	16,767,943	7.1E‐09	0.13	T	A	16.1	FarmCPU
		Ca10	S10_16718457	16,718,457	7.2E‐10	0.07	C	T	3.4	FarmCPU

Abbreviations: Chr, citron watermelon chromosome; MAF, minor allele frequency; PVE, phenotypic variation explained.

**FIGURE 4:**
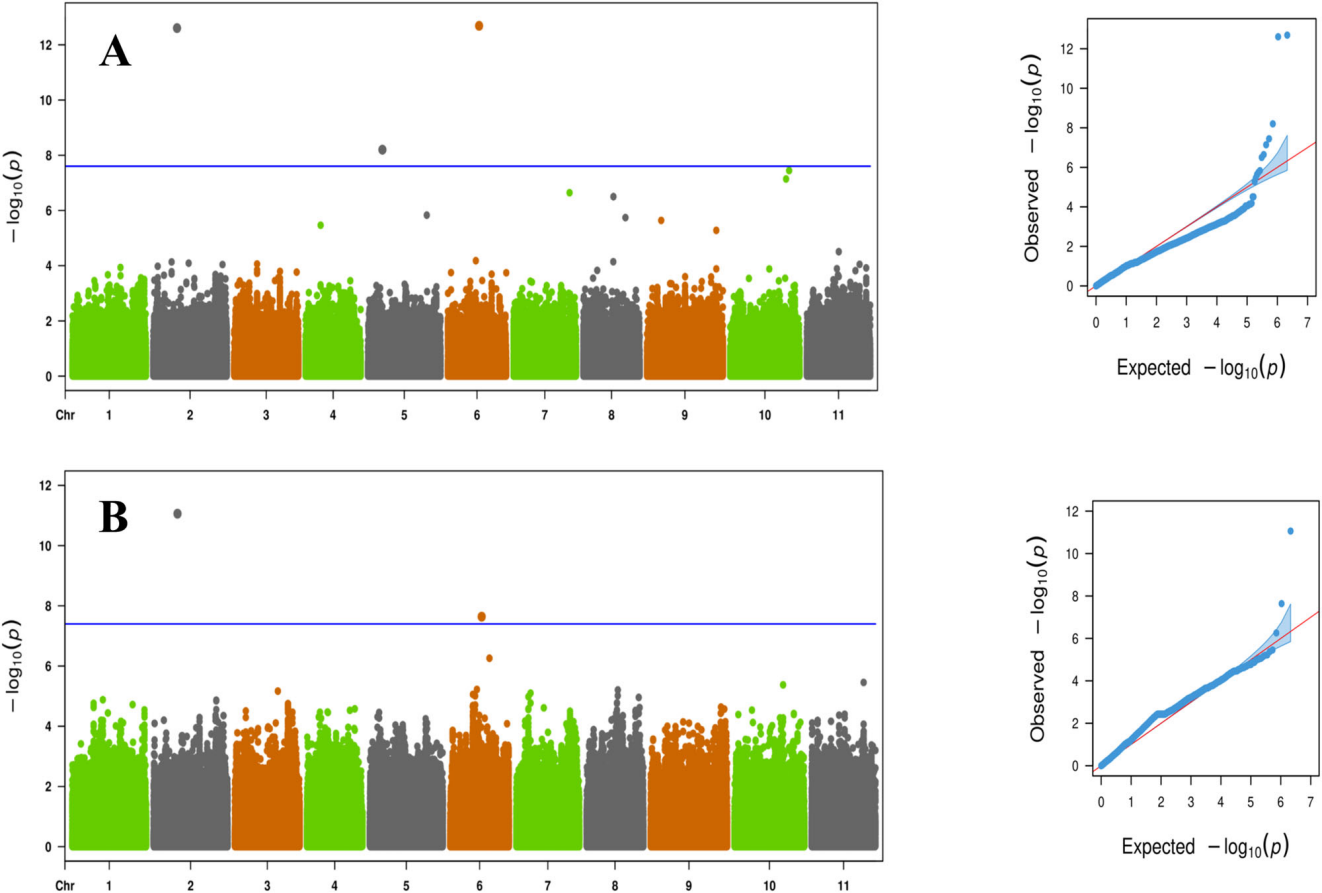
Manhattan and quantile–quantile (QQ) plots for vine length based on (A) FarmCPU (fixed and random model circulating probability unification) and (B) BLINK GWAS, where BLINK is Bayesian‐information and linkage‐disequilibrium iteratively nested keyway and GWAS is genome‐wide association study, models across the 122 cold‐stressed citron watermelon genotypes using best linear unbiased estimates (BLUEs) from the growth chamber screening. The blue solid horizontal line is a false discovery rate (FDR) cutoff of *α* = 0.05.

Three significant SNP markers (*p* value ≤ 6.3 × 10^−9^) were associated with vine length and located on chromosomes Ca02, Ca05, and Ca06 (Table 4; Figure 4A,B). The peak SNPs on chromosomes Ca02 and Ca06 were detected by both BLINK and FarmCPU models (Table 4; Figure 4A,B). These three peak SNPs explained 4.4%–15.7% of the phenotypic variation in vine length (Table 4).

**FIGURE 5:**
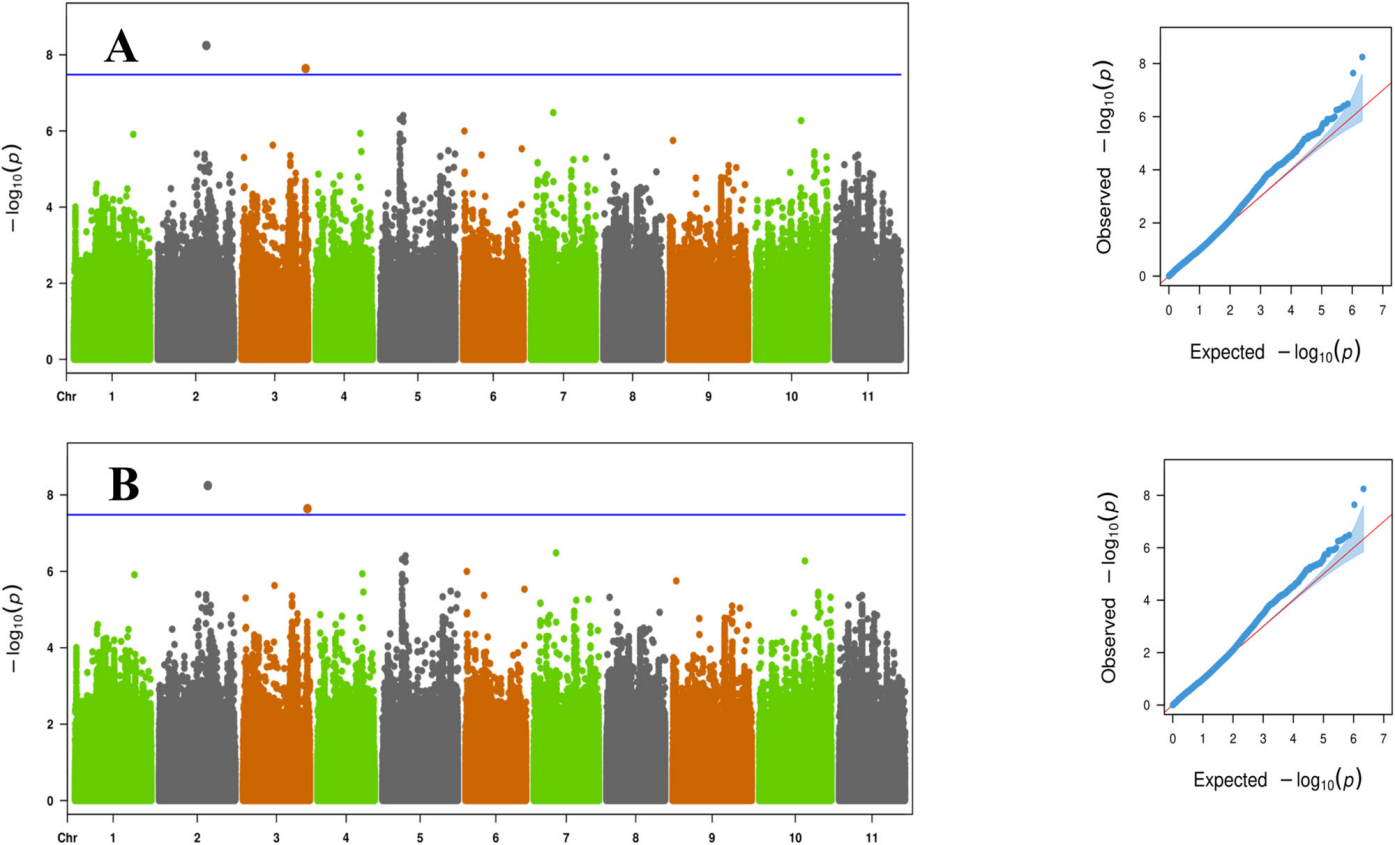
Manhattan and quantile–quantile (QQ) plots for *F_v_/F_m_* based on (A) FarmCPU (fixed and random model circulating probability unification) and (B) BLINK GWAS, where BLINK is Bayesian‐information and linkage‐disequilibrium iteratively nested keyway and GWAS is genome‐wide association study, models across the 122 non‐stressed control citron watermelon genotypes using best linear unbiased estimates (BLUEs) from the growth chamber screening. The blue solid horizontal line is aa false discovery rate (FDR) cutoff of *α* = 0.05.

**FIGURE 6:**
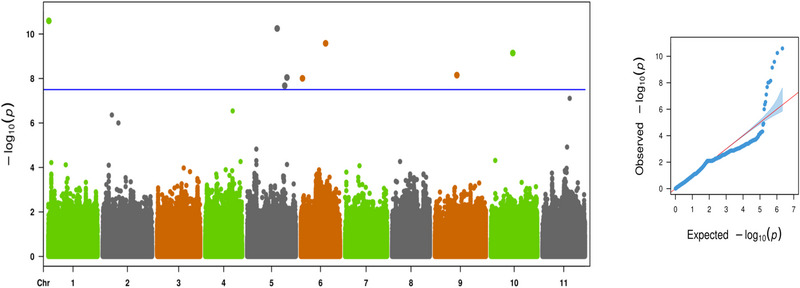
Manhattan and quantile–quantile (QQ) plots for vine length based on FarmCPU GWAS, where FarmCPU is fixed and random model circulating probability unification and GWAS is genome‐wide association study, model across the 122 non‐stressed control citron watermelon genotypes using best linear unbiased estimates (BLUEs) from the growth chamber screening. The blue solid horizontal line is aa false discovery rate (FDR) cutoff of *α* = 0.05.

Under non‐stress control conditions, two significant SNPs for *F_v_*/*F_m_* including S2_24000594 and S3_31686848 on chromosomes Ca02 and Ca03 were each detected by both BLINK and FarmCPU models (Table 4; Figure 5A,B). The SNP markers explained 13.7% and 21.8% of the phenotypic variation in *F_v_*/*F_m_* (Table 4). Eight SNP markers were associated with vine length under non‐stress control conditions (*p* value ≤ 2.1 × 10^−8^) and were located on chromosomes Ca01, Ca05, Ca06, Ca09, and Ca10 (Table 4; Figure 6). These eight peak SNP markers were detected by FarmCPU model and explained 2.5%–16.1% of the phenotypic variation in vine length (Table 4).

**FIGURE 7:**
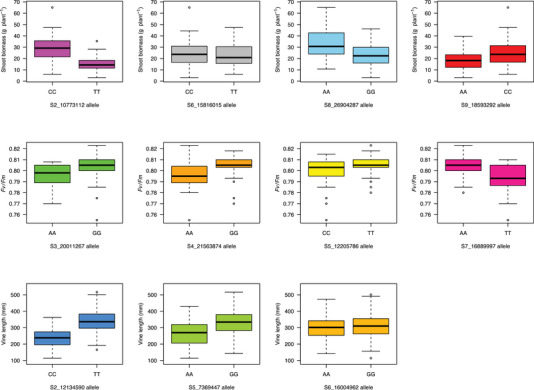
Boxplot showing allelic effects of the significant single nucleotide polymorphism (SNP) markers associated with shoot biomass, *F_v_*/*F_m_*, and vine length traits in the 122 cold‐stressed citron watermelon seedlings based on BLINK (Bayesian‐information and linkage‐disequilibrium iteratively nested keyway) and FarmCPU GWAS, where FarmCPU is fixed and random model circulating probability unification and GWAS is genome‐wide association study, models.

Under cold stress conditions, for shoot biomass, the CC major allele of SNP S2_10773112 on chromosome Ca02 had a higher mean performance of 28.9 g plant^−1^ compared to genotypes homozygous for the TT minor allele whose mean was 15.4 g plant^−1^ (Table 5; Figure 7). Mean allelic shoot biomass values for peak SNP marker S6_15816015 were statistically similar (Table 5; Figure 7). Genotypes carrying the minor allele class of AA had a heavier shoot biomass mean measurement of 33.2 g plant^−1^ compared to individuals with the major allele class of GG with a mean of 23.1 g plant^−1^ (Table 5; Figure 7). The CC major allele of SNP S9_18593292 on chromosome Ca09 had a higher mean shoot biomass of 25.1 g plant^−1^ compared to genotypes homozygous for the AA minor allele whose mean was 19.0 g plant^−1^ (Table 5; Figure 7). Mean allelic *F_v_*/*F_m_* performances for all peak SNPs under cold stress conditions were statistically similar (Table 5; Figure 7). For vine length, two of the three peak SNPs had mean performances that were statistically different (Table 5; Figure 7). On chromosome Ca02 (SNP S2_12134590), genotypes with the TT major allele class had longer vines of 339.4 mm compared to accessions with the CC minor allele class, which had shorter vines with a mean of 234.0 mm (Table 5; Figure 7). For the SNP marker S5_7369447 on chromosome Ca05, genotypes with the major allele class of GG had longer vines with a mean of 330.2 mm compared to accessions with the AA minor allele class that had a mean of 265.8 mm (Table 5; Figure 7).

**TABLE 5:** Allelic effects of the significant single nucleotide polymorphism (SNP) markers associated with shoot biomass, *F_v_*/*F_m_*, and vine length traits in the cold‐stressed and non‐stressed control 122 citron watermelon seedlings based on BLINK (Bayesian‐information and linkage‐disequilibrium iteratively nested keyway) and FarmCPU GWAS, where FarmCPU is fixed and random model circulating probability unification and GWAS is genome‐wide association study, models.

Traits	Treatment	Chr	SNP	Major allele	Minor allele	Mean of major allele	Mean of minor allele	*df*	*t* value	*p* value
Shoot biomass (g plant^−1^)	Cold‐stressed	Ca02	S2_10773112	C	T	28.9	15.4	105.6	8.79	3.0 × 10^−14^
		Ca06	S6_15816015	C	T	24.4	23.0	55.3	0.65	ns
		Ca08	S8_26904287	G	A	23.1	33.2	15.7	2.61	0.0191
		Ca09	S9_18593292	C	A	25.1	19.0	19.7	−2.35	0.0291
*F_v_*/*F_m_*	Cold‐stressed	Ca03	S3_20011267	G	A	0.80	0.79	12.4	−0.55	ns
		Ca04	S4_21563874	G	A	0.81	0.79	21.5	−0.61	ns
		Ca05	S5_12205786	T	C	0.81	0.79	69.3	−0.59	ns
		Ca07	S7_16889997	A	T	0.80	0.79	14.8	0.52	ns
	Non‐stressed	Ca02	S2_24000594	C	T	0.82	0.77	6.1	0.73	ns
		Ca03	S3_31686848	T	A	0.82	0.79	11.4	−0.59	ns
Vine length (mm)	Cold‐stressed	Ca02	S2_12134590	T	C	339.4	234.0	72.6	−8.64	9.1 × 10^−13^
		Ca05	S5_7369447	G	A	330.2	265.8	76.2	−4.42	3.3 × 10^−5^
		Ca06	S6_16004962	G	A	309.9	298.3	54.0	−0.71	ns
	Non‐stressed	Ca01	S1_451332	A	C	382.8	436.9	97.2	−3.48	7.4 × 10^−4^
		Ca05	S5_22850438	G	A	412.1	347.8	19.0	−3.61	1.8 × 10^−3^
		Ca05	S5_30282239	T	C	397.2	413.8	118.9	1.08	ns
		Ca06	S6_18850800	C	T	437.8	367.4	111.4	5.17	1.1 × 10^−6^
		Ca09	S9_16767943	T	A	403.7	414.6	19.1	0.45	ns
		Ca10	S10_16718457	C	T	398.9	461.8	12.1	−1.83	ns

Abbreviations: *df*, degrees of freedom; ns, not significant.

**FIGURE 8:**
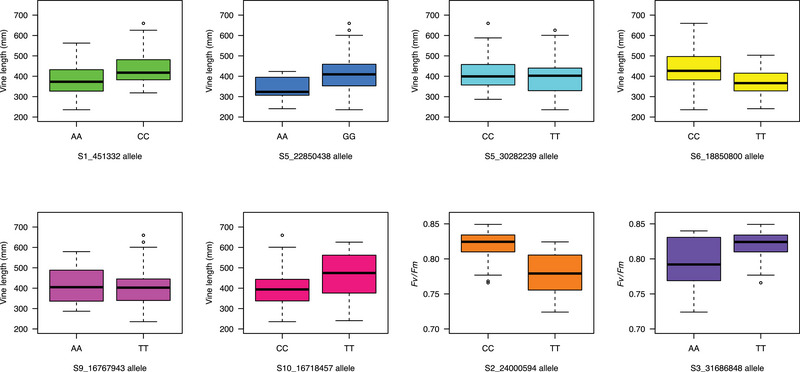
Boxplot showing allelic effects of the significant single nucleotide polymorphism (SNP) markers associated with vine length and *F_v_*/*F_m_* traits in the non‐stressed control 122 citron watermelon seedlings based on BLINK (Bayesian‐information and linkage‐disequilibrium iteratively nested keyway) and FarmCPU GWAS, where FarmCPU is fixed and random model circulating probability unification and GWAS is genome‐wide association study, models.

Under non‐stress control conditions, mean allelic *F_v_*/*F_m_* performances for both peak SNP markers were statistically similar (Table 5; Figure 8). Three peak SNPs associated with vine length under the non‐stress control conditions had statistically different allelic means (Table 5; Figure 8). On chromosome Ca01, genotypes with allele AA for SNP S1_451332 had shorter vine length mean of 382.8 mm compared to those with allele CC, which had a mean vine length of 436.9 mm under non‐stress control conditions (Table 5; Figure 8). The allele GG of SNP marker S5_22850438 on chromosome Ca05 was associated with longer vines (mean = 412.1 mm) compared to genotypes with allele AA, which had a mean vine length of 347.8 mm (Table 5; Figure 8). Genotypes carrying the CC allele of SNP marker S6_18850800 on chromosome Ca06 had a higher mean vine length of 437.8 mm compared to the TT allele with a mean of 367.4 mm (Table 5; Figure 8).

### 3.3 Candidate genes

Four classes of candidate genes relevant to low‐temperature stress tolerance and photosynthesis co‐located with the peak SNPs on chromosomes Ca01, Ca03, Ca04, Ca05, and Ca08. For shoot biomass under cold stress conditions, two candidate genes (*CaU08G17330* and *CaU08G17440*) co‐located with the peak SNP S8_26904287 and encode for calmodulin proteins. Candidate gene *CaU08G17330* was located 31.7 Kb upstream of the peak SNP S8_26904287. Candidate gene *CaU08G17440* was located 56.1 Kb downstream of the peak SNP S8_26904287. For *F_v_*/*F_m_* under cold stress conditions, two candidate genes (*CaU04G07240* and *CaU04G07290*) co‐located with peak SNP S4_21563874 on chromosome Ca04 and code for an ethylene‐responsive transcription factor and an abscisic acid receptor, respectively. Candidate gene *CaU04G07240* was located 36.2 Kb upstream of the peak SNP S4_21563874. Candidate gene *CaU04G07290* was located 20.6 Kb downstream of the peak SNP S4_21563874. Candidate gene *CaU05G14340* was located at 47.7 Kb upstream of the peak SNP S5_12205786 on chromosome Ca05 and codes for a zinc finger protein.

Under non‐stress control conditions for *F_v_*/*F_m_*, candidate gene *CaU03G19760* was found at 70.2 Kb downstream of the peak SNP S3_31686848 on chromosome Ca03 and codes for calmodulin proteins. The SNP marker S1_451332 associated with vine length co‐located with three candidate genes. Candidate gene *CaU01G00590* was found at 57.5 Kb upstream of the SNP S1_451332 and codes for a zinc finger protein. Candidate gene *CaU01G00690* was found at 16.9 Kb downstream of the SNP S1_451332 and codes for an abscisic acid receptor. Candidate gene *CaU01G00790* was found at 89.1 Kb downstream of the SNP S1_451332 and codes for an ethylene‐responsive transcription factor. Candidate gene *CaU05G22070* was found at 50.7 Kb downstream of the peak SNP S5_28595908 on chromosome Ca05 and codes for a zinc finger protein. Candidate gene *CaU05G24320* was found at 40.6 Kb downstream of the peak SNP S5_30282239 on chromosome Ca05 and codes for calmodulin proteins.

## 4 DISCUSSION

### 4.1 Genotype performance, broad‐sense heritability, and correlation analysis

Watermelon is an economically important annual vegetable crop of tropical origin (Chomicki et al., 2020; Chomicki & Renner, 2015). The crop is sensitive to low‐temperature stress and suffers from chilling injury when transplanted at 4 weeks to the open field in early spring in temperate production regions (Bradow, 1990; Korkmaz & Dufault, 2001). Active screening of watermelon germplasm and genetic mapping strategies to identify genotypes and genomic regions for tolerance to low‐temperature stress will be central to the breeding efforts aimed at development of resilient watermelon varieties. Significant differences among the *C*. *amarus* genotypes for the evaluated low‐temperature stress related traits in this research indicate that parental selection to create recombinants and introgress the tolerance alleles through traditional plant breeding is feasible.

This research revealed the presence of a genetic influence for all traits (shoot biomass, vine length, *F_v_*/*F_m_*, and chlorophyll content) in the citron watermelon germplasm collection. This genetic variation allowed for identification of superior germplasm for the four evaluated traits. Genotypes USVL_114 and PI_532664 had high values for both shoot biomass and vine length, while accession PI_482246 had high chlorophyll content. These genotypes can be targeted in parental selections to improve watermelon for low‐temperature stress tolerance. Root traits in sorghum and watermelon have been shown to play an important role in the plants’ ability to cope with cold stress conditions (Bradow, 1990; Chopra et al., 2017). Interestingly, genotype PI_482246 with high chlorophyll content in this research also performed above the population mean for total root length in a large watermelon root traits screening study (Katuuramu et al., 2020). Overall, comparative analysis revealed lower trait means within the cold‐stressed plants compared to the non‐stressed control plants in this research. Low‐temperature stress has been reported to cause various physiological and metabolic disorders including impairment of the photosynthetic machinery across many plant species, ultimately inhibiting normal growth and production (Bradow, 1990; Revilla et al., 2005; Shinozaki et al., 2015).

Broad‐sense heritability for the evaluated traits ranged from moderately low to high (0.35–0.73), indicating the presence of inherent genetic control for low‐temperature stress tolerance in the *C*. *amarus* collection. As low‐temperature stress tolerance is important for watermelon varieties grown in colder climates around the world, these empirical observations on native variation for cold stress tolerance offer a great opportunity to improve watermelon through breeding and selection. Trait correlation analysis is used to determine positive and negative associations among phenotypic traits, identify parental combinations useful in cultivar development, and reduce trait measurement redundancy (Yan & Fregeau‐Reid, 2008). Correlation analysis revealed the presence of positive relationships among all trait combinations. Positive correlations between chlorophyll content and *F_v_*/*F_m_* have also been previously reported in other crops such as maize evaluated under low‐temperature growth conditions (Revilla et al., 2014). The occurrence of positive correlations among the evaluated traits suggests that these four phenotypes can be targeted concurrently to improve watermelon for low‐temperature stress tolerance.

### 4.2 Marker‐trait associations and candidate genes

To date, there has been limited research efforts toward understanding the genetic control for low‐temperature stress tolerance in watermelon. A few of these studies have focused on screening method optimization and worked with small sets of watermelon genotypes (Bradow, 1990; Kozik & Wehner, 2014; Korkmaz & Dufault, 2002). In the present study, significant marker‐trait associations were identified for shoot biomass, vine length, and *F_v_*/*F_m_* on chromosomes Ca02, Ca04, Ca05, Ca06, and Ca08. The identification of SNP markers associated with low‐temperature stress tolerance traits in this research will be useful in the development of watermelon varieties for colder growing regions via marker‐assisted breeding.

Tolerance to low‐temperature stress in plants is a complex trait controlled by multiple genes and transcription factors (Hou et al., 2018; Song et al., 2018; Yagcioglu et al., 2019). Molecular networks for low‐temperature stress sensing appear to be largely conserved across plants (Knight & Knight, 2012). Plant response to low‐temperature stress involves induction of genes and mechanisms for signal transduction such as abscisic acid, carbon assimilation, photosynthetic efficiency, and lipid composition (Janska et al., 2010; Kaniuga et al., 1999; Sobkowiak et al., 2016, 2014). In this research, four classes of candidate genes (calmodulin protein, zinc finger protein, ethylene‐responsive transcription factor, and abscisic acid receptor) co‐located with the peak SNPs for shoot biomass, maximum quantum efficiency of photosystem II (*F_v_*/*F_m_*), and vine length. Calmodulins are plant sensor proteins involved in the regulation of developmental processes and plant's response to environmental cues and abiotic stressors including cold stress tolerance (Miura & Furumoto, 2013; Perochon et al., 2011; Zeng et al., 2015). Zinc finger proteins are important during plant growth and development and play key roles in the response to abiotic stress such as low‐temperature stress (Shi et al., 2014). X. Wang et al. (2018) reported a stress‐associated locus *OsSAP16* encoding for zinc finger protein to be responsible for low‐temperature tolerance during germination in rice. *Arabidopsis thaliana* mutants with extremely low levels of ethylene production were hypersensitive to low‐temperature stress (Catala et al., 2014). Working with a cucumber biparental cross between low‐temperature tolerant and low‐temperature sensitive genotypes, Dong et al. (2019) validated an ethylene‐responsive transcription factor as one of the candidate genes within the genomic region controlling low‐temperature stress tolerance. Abscisic acid (ABA) is an important phytohormone that regulates essential physiological and biochemical processes during growth and development and is a key player in the plants’ response to abiotic stressors such as low‐temperature stress (Kim et al., 2016; Kumar et al., 2008). Under low‐temperature stress conditions, there was an upregulation in the expression of ABA responsive receptors in *A*. *thaliana* (Choi et al., 2000). Additionally, overexpression of an ABA receptor gene (*VaPYL4*) from cold‐hardy grape plants in *A*. *thaliana* resulted in enhanced tolerance to low‐temperature stress (Ren et al., 2022).

## 5 CONCLUSION

In this study, 122 USDA citron watermelon germplasm accessions were phenotyped for four low‐temperature stress tolerance related traits including shoot biomass, vine length, *F_v_*/*F_m_*, and chlorophyll content. Genome‐wide association analysis mapping methodology was deployed to detect marker‐trait associations. The significant SNP markers and candidate genes uncovered in this research provide valuable resources for future studies seeking to enhance the understanding of the genetic underpinnings for low‐temperature stress tolerance and genetically improve watermelon through marker assisted breeding. The low‐temperature tolerant genotypes can be targeted in parental selections to develop resilient watermelon varieties for production in colder climates.

## AUTHOR CONTRIBUTIONS

**Dennis N. Katuuramu**: Conceptualization; methodology; investigation; data curation; formal analysis; visualization; writing—original draft; writing—review and editing. **Amnon Levi**: Conceptualization; resources; writing—review and editing. **William P. Wechter**: Conceptualization; resources; writing—review and editing.

## CONFLICT OF INTEREST STATEMENT

The authors declare no conflicts of interest.

## Data Availability

The original contributions presented in this research are available within the article. The genomic datasets used in this research can be accessed at different websites (http://cucurbitgenomics.org/; http://cucurbitgenomics.org/ftp/genome/watermelon/USVL246/; and http://cucurbitgenomics.org/ftp/reseq/watermelon/Amarus_reseq/). Any reasonable inquiries/requests can be made to the corresponding author.
